# A comparison study of posterior cervical percutaneous endoscopic ventral bony decompression and simple dorsal decompression treatment in cervical spondylotic radiculopathy caused by cervical foraminal and/or lateral spinal stenosis: a clinical retrospective study

**DOI:** 10.1186/s12891-020-03313-2

**Published:** 2020-05-11

**Authors:** Yuexin Tong, Zhangheng Huang, Chuan Hu, Zhiyi Fan, Fucheng Bian, Fengkai Yang, Chengliang Zhao

**Affiliations:** 1 Department of Spine Surgery, Affiliated Hospital of Chengde Medical University, Shuangqiao District, Chengde, Hebei Province China; 2 Department of Orthopedic Surgery, The Affiliated Hospital of Qingdao University, Shinan District, Qingdao, Shandong province China

**Keywords:** Percutaneous endoscopic cervical decompression (PECD), Cervical foraminal and/or lateral spinal stenosis (CFa/oLSS), Cervical spondylotic radiculopathy (CSR), Posterior percutan-eous cervical endoscopic decompression-ventral bony decompression (PPECD-VBD), Posterior percutaneous cervical endoscopic decompression-simple dorsal decompression (PPECD-SDD), Minimally invasive surgery

## Abstract

**Background:**

Percutaneous endoscopic cervical decompression (PECD) is an ideal minimally invasive decompression technique for the treatment of cervical spondylotic radiculopathy (CSR). However, the mainstream is the resection of dorsal bone and removal of free nucleus pulposus. The necessity of excision of ventral osteophytes and hyperplastic ligaments in the treatment of CSR caused by cervical foraminal and/or lateral spinal stenosis (CFa/oLSS) to be discussed.

**Methods:**

We performed a retrospective study of 46 patients with CSR caused by CFa/oLSS from January 2017 to November 2018. These patients received posterior percutaneous endoscopic cervical decompression-ventral bony decompression (PPECD-VBD)(23 cases, classified as VBD group) or posterior percutaneous endoscopic cervical decompression-simple dorsal decompression (PPECD-SDD)(23 cases, classified as SDD group). Following surgery, we recorded Visual Analogue Scale (VAS), Neck Disable Index (NDI), Japanese Orthopaedic Association (JOA) Scores and myodynamia. We further evaluated the changes of cervical curvature and cervical spine motion in the VBD group and recorded the operation time and complications during the follow-up of each patient.

**Results:**

All patients underwent successful operations, with an average follow-up time of 16.53 ± 9.90 months. The excellent and good rates in the VBD and SDD groups were 91.29 and 60.87%, respectively. In the SDD group, neck-VAS, arm-VAS, and NDI scores were significantly higher than those of the VBD group at 1 day, 6 months, and 12 months after surgery (*P* < 0.05), while the JOA scores and improvement rate of JOA were significantly lower than those of the VBD group (P < 0.05). There were no significant differences in terms of angular displacement (AD), horizontal displacement (HD), segmental angle (SA) and cervical curvature (CA) before and after the operation in the VBD group (*P* > 0.05).

**Conclusion:**

PPECD-VBD was significantly better than PPECD-SDD as well as PPECD-VBD had no significant effects on cervical spine stability or cervical curvature.

## Background

Cervical spinal stenosis is a type of spinal disease caused by spinal cord or nerve root compression resulting from various types of reduced spinal canal space. Cervical spondylotic radiculopathy (CSR) occurs when stenosis occurs in the intervertebral foramen or lateral spinal canal, compressing the cervical nerve root [1]. Surgical treatment is feasible when conservative treatment cannot relieve clinical symptoms [2].

The anterior cervical discectomy and fusion (ACDF) pioneered by Robinson and Smith [3] is considered to be the “gold standard” for CSR [4, 5]. This is because the anterior approach can more completely remove nerve ventral osteophytes, hyperplastic ligaments, protruding discs, and other pressure substances to achieve thorough ventral decompression, thereby fully releasing the compressed nerves [6–10]. Although the anterior approach is generally accepted by spine surgeons, it has certain limitations, such as limited neck movement, degeneration of adjacent segments, ectopic ossification and mechanical failure, high reoperation rates, and other complications [11–14].

Posterior cervical foraminotomy (PCF), which was first described by Spurling and Scoville [15], is another classic treatment for CSR caused by cervical foraminal and/or lateral spinal stenosis (CFa/oLSS). Indirect decompression of the cervical nerve root is achieved by removing the medial part of the facet joint and expanding the foramen to relieve root symptom [16]. Percutaneous endoscopic cervical decompression (PECD) was developed based on PCF [17]. Quillo-Olvera reported that PECD is an effective treatment for CSR caused by cervical disc herniation [18]. PECD is mainly used for the treatment of CSR by dorsal decompression of the nerve root and excision of the free disc in the surgical area without removal of neuroventral osteophytes or hyperplastic ligaments [19–23]. It is worth mentioning that the pressure substances of CSR caused by CFa/oLSS also include ventral osteophytes, calcified discs, and hyperplastic ligaments; disc herniation is also included in some cases. Thus, the decompressive effect of PECD is not as good as ACDF. This is similar to microtomy foraminal incision. This may be the reason there is no significant difference in the efficacy of PECD and microendoscopic posterior cervical foraminotomy [24].

Oh Hyeong Seok et al. reported that 12 (11.9%) of 101 CSR patients who received PECD experienced poor clinical efficacy; five patients combined CFa/oLSS among them [25]. That is to say, the currently mainstream PECD (we defined it as posterior percutaneous endoscopic cervical decompression-simplel dorsal decompression (PPECD-SDD) in the present study) may has some deficiency in the treatment of CSR caused by CFa/oLSS. After a large number of patients received posterior percutaneous cervical endoscopic surgery, we noticed that patients’ satisfaction was generally higher after resection of ventral osteophyte and thickening ligament than those without preceding steps. On the basis of above, we first proposed the theory of posterior percutaneous endoscopic cervical decompression-ventral bony decompression (PPECD-VBD) for CSR caused by CFa/oLSS. In theory, compared with PPECD-SDD, the technique of PPECD-VBD included the resection of ventral osteophytes, hyperplastic ligaments on the basis of PPECD-SDD, which meant more thorough ventral decompression and more significant clinical outcome. However, there is no relevant literatures reported whether the curative effectiveness of PECD combined resection of ventral osteophytes, hyperplastic ligaments in the treatment of CSR caused by CFa/oLSS is better or not currently. For answering this question, we performed a retrospective study to explore the difference in efficacy between PPECD-VBD and PPECD-SDD, which may have important significance for standardizing the decompression standard of posterior cervical percutaneous endoscope for CSR caused by CFa/oLSS.

## Methods

### Study design

This was a retrospective cohort study from January 2017 to November 2018. All the data were analyzed anonymously. An informed consent waiver was granted.

### Patients

Patients in both groups strictly followed the inclusion and exclusion criteria. Inclusion criteria were as follows: (1) clear diagnosis of CFa/oLSS; (2) ineffective conservative treatment for at least 3 months; (3) unilateral root symptoms, such as pain, numbness, weakness in the affected limb with or without neck pain consistent with the diagnosis results from magnetic resonance imaging (MRI) and computed tomography (CT); and (4) preoperative diagnosis based on MRI and CT showed ventral pressure compressive substances such as ventral osteophytes, disc calcification, and ligament hypertrophy. Exclusion criteria were as follows: (1) pure “soft” cervical disc herniation; (2) clear diagnosis of cervical spondylotic myelopathy; (3) cervical infection or tumor; and (4) neck pain or upper arm pain numbness, weakness caused by other causes.

During the study periods, a total of 62 consecutive patients who underwent posterior cervical percutaneous endoscopic nerve root decompression were identified from our surgery team. Among the 62 patients, 16 patients were excluded (14 cases for reason (1), 2 cases for reason (2)). Thus, 46 patients were finally enrolled in this study, of which 23 patients received PPECD-VBD treatment (classified as VBD group) while the others (23 patients) received PPECD-SDD (classified as SDD group).

### Surgical technique

#### SDD group

The patients in the SDD group received local anesthesia and intravenous composite general anesthesia; patients were conscious with no tracheal intubation. The patient was lying prone on the position frame, with the head and cervical vertebra supported by a headstock, and the neck was slightly forward-flexed. The vertebral space of the lesion was determined by fluoroscopy. The incision was 3 cm from the midline located on the affected side. Then, 30 mL of 0.8% lidocaine was sequentially subcutaneously anesthetized by subcutaneous tissue, deep fascia, dorsal side of joint and capsule of the articular process joint. An lateral skin incision of 8 mm was made parallel to the joint space. A soft tissue working channel was gradually established, and a working sleeve was further inserted through a dilator. When visualizing the working sleeve was located at joint space and the inner edge of the pedicle projection, it was connected to an endoscope. Facet joints and lamina soft tissue were removed by grasping forceps and a bipolar RF electrode under the microscope, revealing the “V” point. The upper and lower edges of the adjacent vertebral plate were removed with a microscope drill to expose the yellow ligament moving towards the medial side of the facet joint. Following excision of the bone structure and the ligamentum flavum, the outer edge of the dural sac and the dorsal side of the nerve root were exposed. The nerve root and the ventral side of the outer edge of the spinal cord were explored. While free nucleus pulposus might need to be removed, the ventral osteophytes, hyperplastic ligaments, and inclusive disc organization were left intact. The incision was sutured after drainage was placed.

#### VBD group

**Fig. 1:**
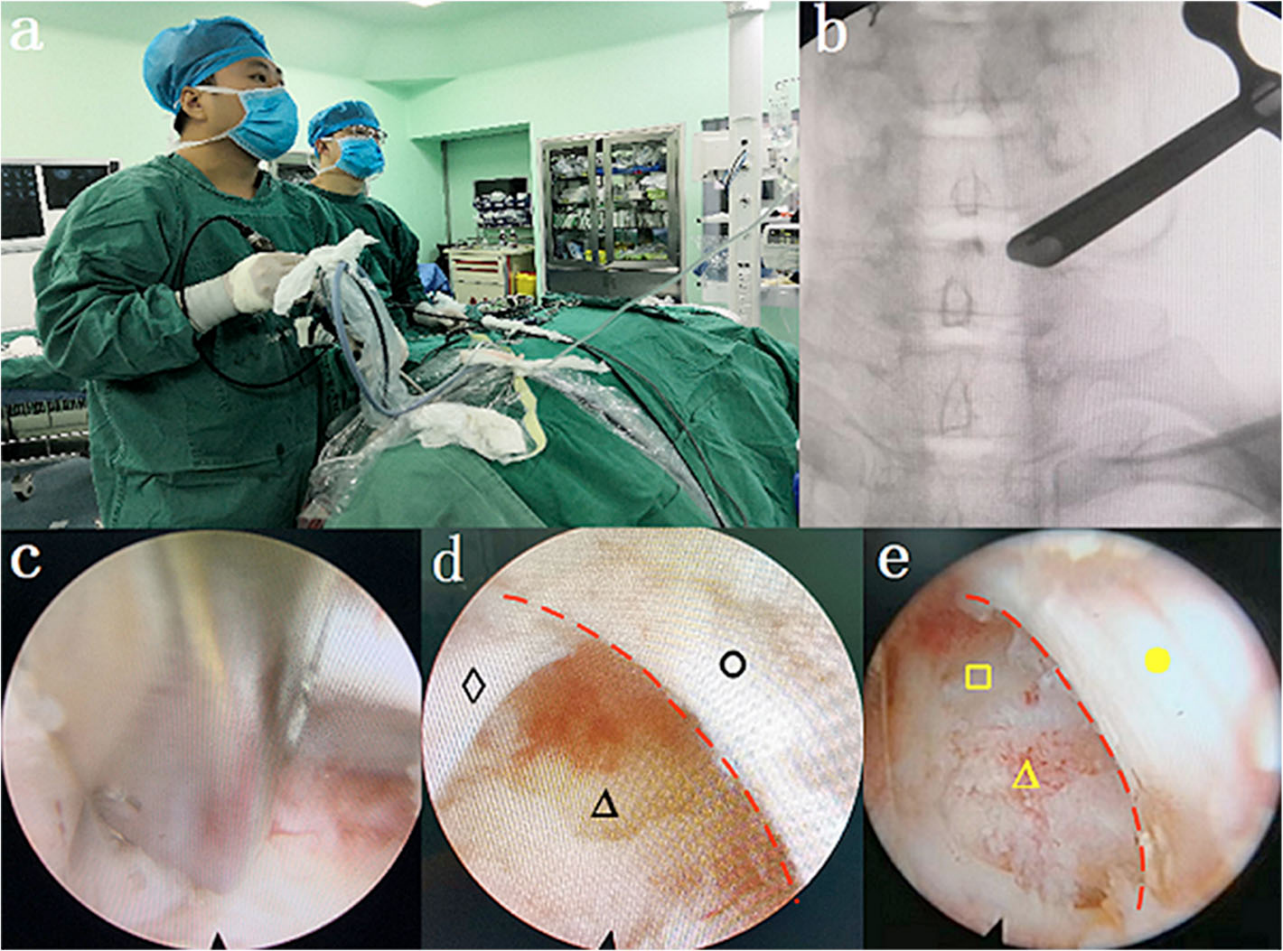
Surgical procedure.**a** Surgeon during operation.**b** The position of the working sleeve.**c** Removal of ventral osteophytes. Both **d** and **e** Spinal cord is represented by yellow and black circle, respectively. Vertebral edge with ventral osteophytes removed is represented by yellow triangle, ventral pressure substances is represented by black triangle, nerve root is represented by black diamond

Surgical procedures before revealing the “V” point are the same as the SDD group. The procedure differ beginning with grinding the interlaminar foramen. The scope of the interlaminar foramen was slightly expanded outward and the tail side until fully revealing the axillary area of the nerve root, and the working sleeve could fall into the axilla. This allowed the cannula and endoscope to have adequate abduction angles to facilitate exploration and resection of the outer spinal cord and nerve root ventral osteophytes, hyperplastic ligaments and inclusive disc herniation. In some cases, ventral osteophytes were removed with nucleus pulposus forceps, a curved osteotome, and holmium laser. The operation could not be completed until there was no compression on the ventral side of nerve roots and dural sac in the image of endoscopic as well as the patient’s pain and numbness were significantly relieved. Subsequent procedure were done as described for SDD group (Fig. 1).

### Follow-up

**Fig. 2:**
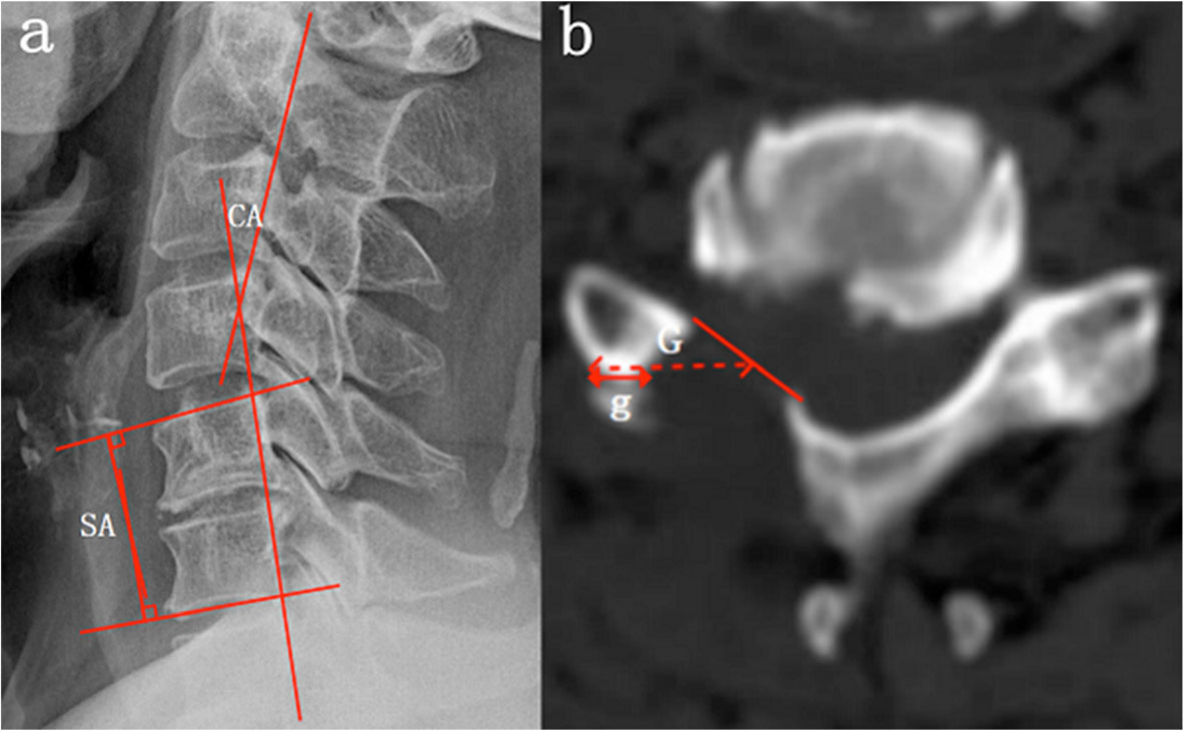
Radiological evaluation. **a** The cervical curvature (CA) is measured using the tangential method from C2 to C7. The segmental angle (SA) is measured the angle between the superior endplate to the inferior endplate of the cephalic and caudal vertebra using Cobb’s method. **b** the ratio of facet joint grinding is calculated as 100% × (G - g)/G

Arm and neck visual analogue scale (VAS), neck disable index (NDI), Japanese Orthopaedic Association (JOA) Scores and myodynamia were recorded to evaluate postoperative efficacy at different time points (before surgery, and 1 day, 6 months, and 12 months after surgery) by a structured telephone interview or returning to hospital. The excellent and good rates was determined by the improved Macnab efficacy evaluation standard at the last follow-up. MRI and CT results were compared before and after surgery. The angular displacement (AD)、horizontal displacement (HD)、segmental angle (SA) and cervical curvature (CA) of the cervical spine and the ratio of facet joint grinding (Fig. 2) of VBD group were measured to evaluate the curvature and stability of the cervical spine. The operation time and complications in both groups were also recorded.

### Statistics

Patients in the two groups were in normal distribution according to the normality test. Two-sample *t*-tests were used to compare efficacy indicators (VAS, NDI, JOA scores and myodynamia) of different time points between two groups, and paired *t*-tests were used to compare the stability and physiological curvature of the cervical spine in the VBD group before and after surgery. The difference of sex, level and side between two groups were analyzed by Chi square test with continuous correction. Fisher’s exact test was used to test the improvement rate of JOA in the two groups. All data were analyzed by SPSS software and displayed as mean ± standard deviation. When the *P* value was less than 0.05, the data was considered statistically significant.

## Results

### Clinical results

**Fig. 3:**
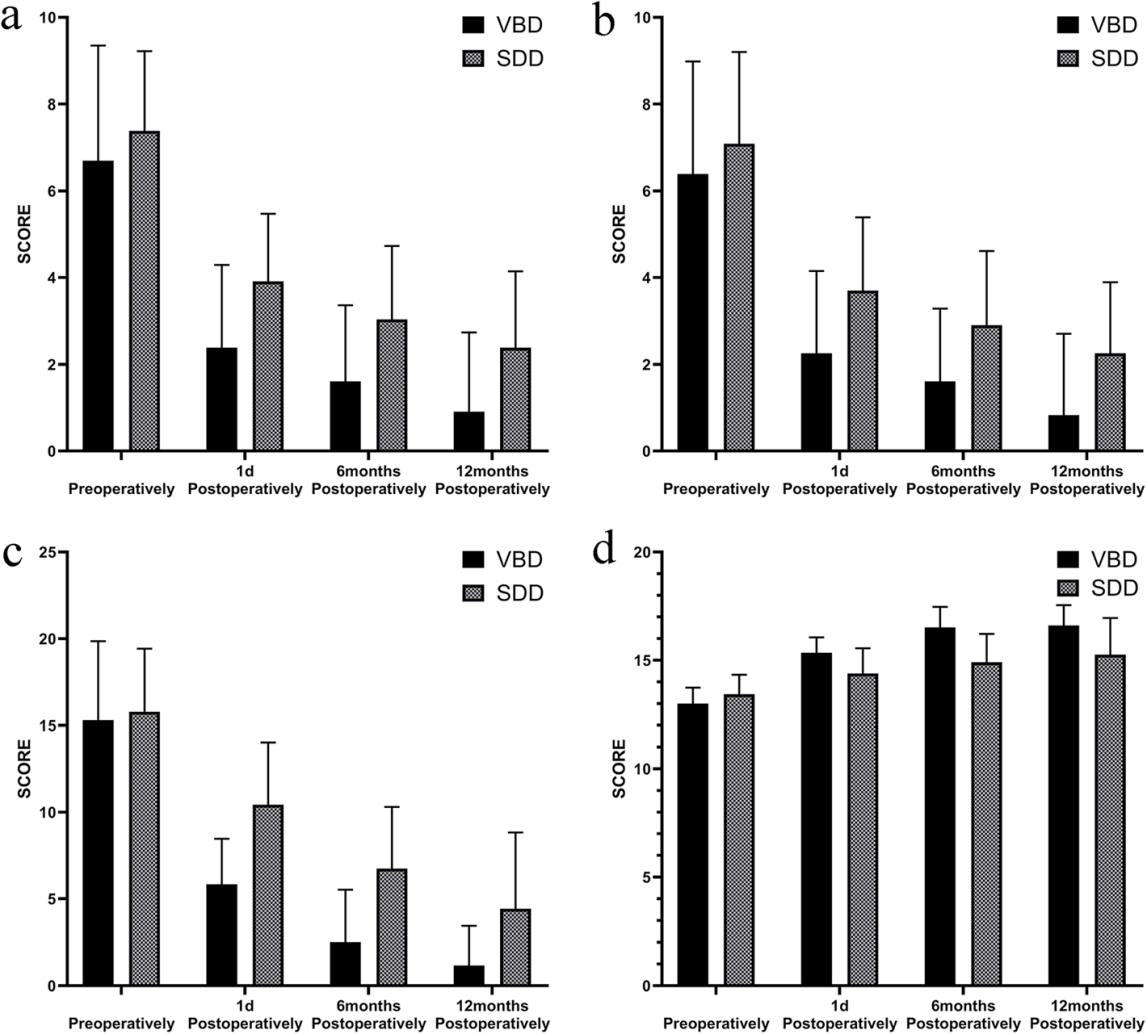
Clinical score in following up. **a** arm-VAS. **b** neck-VAS. **c** NDI score. **d** JOA score

The clinical characteristics of patients in two groups were shown in Table 1. As we saw, all the collected parameters were comparable between two groups. The postoperative follow-up results were shown in Fig. 3 and Table 2 The average operation time was 129.39 ± 9.96 mins in the VBD group and 97.65 ± 7.54 mins in the SDD group. There was no significant intraoperative bleeding in either group. According to the improved Macnab efficacy evaluation standard, the clinical excellent and good rates were 91.29% in the VBD group (11 cases, excellent; 10, good; 1, fair; and 1, poor) and 60.87% in the SDD group (4 cases, excellent; 10, good; 6, fair; and 3, poor). The arm-VAS, neck-VAS, and NDI scores in the VBD group were significantly lower than those of the SDD group at 12 months after surgery. The JOA score of the VBD group was significantly higher than that of the SDD group at 12 months after surgery. These differences were statistically significant (*P* < 0.05). There was no statistical significance in deference of myodynamia between two groups at 12 months after surgery(*P* = 0.15). One patient in the SDD group relapsed 2 years after surgery, and three other patients in the same group had no relief from pain and numbness in the upper extremities and neck. Among the former-mentioned three patients, one received ACDF treatment and corresponding clinical symptoms disappeared thereafter. Moreover, another patient in the SDD group had weak arms after surgery. Only one case of neck pain and poor recovery of upper limb numbness in the VBD group was reported. There were no other complications in follow-up period. Patients in the VBD group had a significantly higher JOA improvement rate at 1 day, 6 months, and 1 year after surgery than the SDD group. The Fisher test between the two groups indicated that the difference was statistically significant.

**Table 1:** Characteristics of patients

	VBD (*N* = 23)	SDD (*N* = 23)	*P* value^a^
Sex, n (%)			
Male	12 (52.2)	9 (39.1)	0.554
Female	11 (47.8)	14 (60.9)	
Level, n (%)			
C 5–6	17 (73.9)	20 (87.0)	0.457
C 6–7	6 (26.1)	3 (13.0)	
Side, n (%)			
Left	12 (52.2)	16 (69.6)	0.365
Right	11 (47.8)	7 (30.4)	
Age, mean ± sd	54.22 ± 10.52	57.48 ± 7.80	0.239
Preop Arm-VAS, mean ± sd	6.70 ± 2.70	7.39 ± 1.83	0.306
Preop Neck-VAS, mean ± sd	6.39 ± 2.59	7.09 ± 2.11	0.323
Preop NDI, mean ± sd	15.30 ± 4.55	15.78 ± 3.64	0.696
Preop JOA, mean ± sd	13.00 ± 0.74	13.43 ± 0.90	0.079
Preop Myodynamia, mean ± sd	4.43 ± 0.59	4.61 ± 0.58	0.32

^a^ Between each item values of two groups

**Table 2:** Clinical outcomes

	Preoperatively	1 day Postoperatively	6 months Postoperatively	12 months Postoperatively	*P* value^a^
**VBD**					
Arm-VAS	6.70 ± 2.70	2.39 ± 1.90	1.61 ± 1.75	0.91 ± 1.83	–
Neck-VAS	6.39 ± 2.59	2.26 ± 1.88	1.61 ± 1.67	0.83 ± 1.88	–
NDI	15.30 ± 4.55	5.83 ± 2.64	2.52 ± 3.00	1.17 ± 2.27	–
JOA	13.00 ± 0.74	15.35 ± 0.71	16.52 ± 0.95	16.61 ± 0.94	–
Improvement rate of JOA	–	58.75%	88.00%	90.25%	–
Myodynamia	4.43 ± 0.59	–	–	5.00 ± 0.00	–
**SDD**					
Arm-VAS	7.39 ± 1.83	3.91 ± 1.56	3.04 ± 1.69	2.39 ± 1.75	0.008
Neck-VAS	7.09 ± 2.11	3.70 ± 1.69	2.91 ± 1.70	2.26 ± 1.63	0.008
NDI	15.78 ± 3.64	10.43 ± 3.59	6.74 ± 3.56	4.43 ± 4.40	0.003
JOA	13.43 ± 0.90	14.39 ± 1.16	14.91 ± 1.31	15.26 ± 1.69	0.002
Improvement rate of JOA	–	27.90%	41.46%	51.26%	0.001
Myodynamia	4.61 ± 0.58	–	–	4.91 ± 0.29	0.15

^a^ Between each item values of two groups at postop 12 months

### Radiological evaluation

**Fig. 4:**
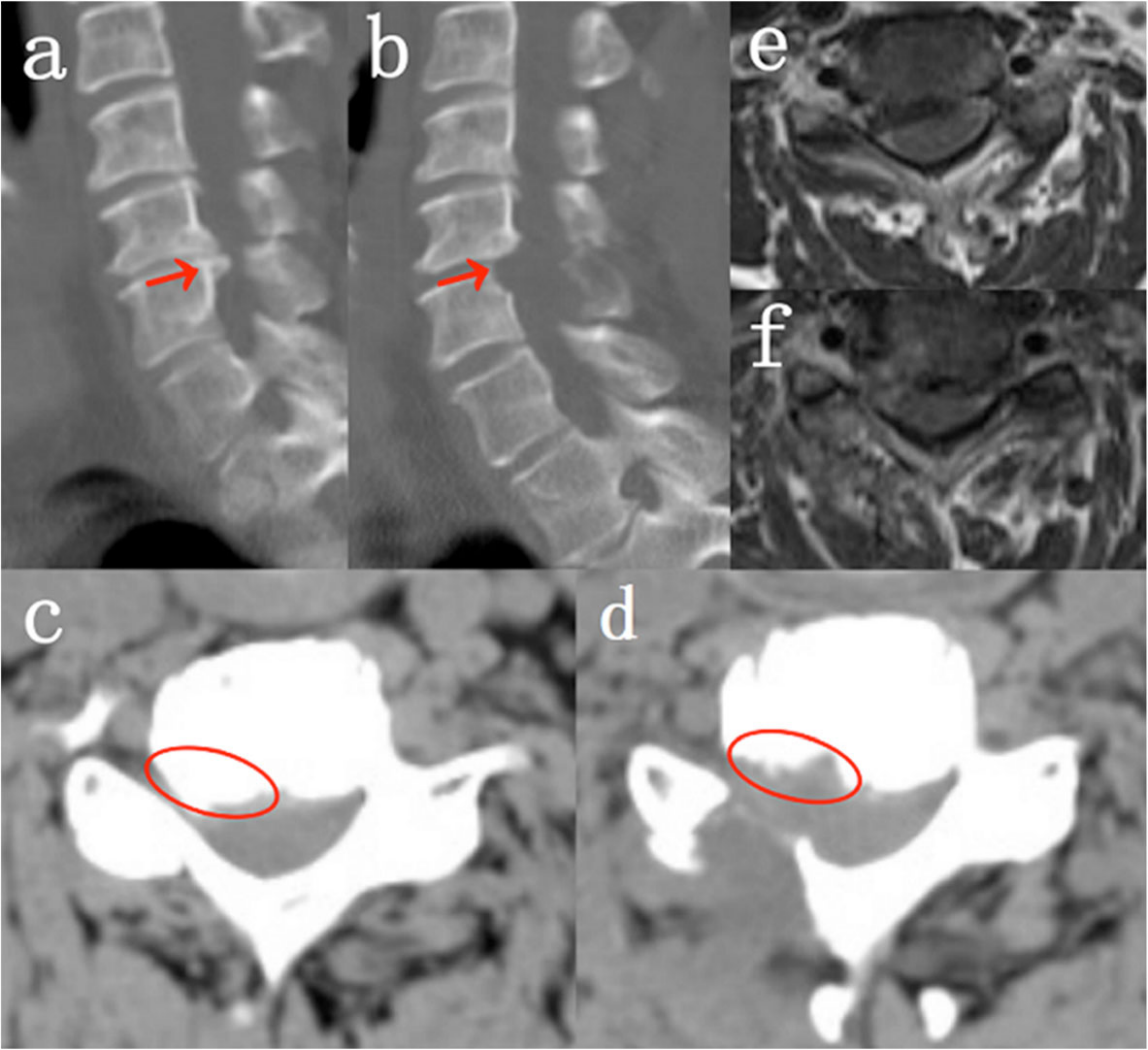
Preoperative and postoperative radiological characteristics of PPECD-VBD. **a** Preoperative CT sagittal image.**b** Postoperative CT sagittal image. **c** Preoperative CT axial image of the surgical segment. **d** Postperative CT axial image of the surgical segment. **e** Preoperative MRI axial image of the surgical segment. **f** Postperative MRI axial image of the surgical segment

Radiological datas of the VBD group were shown in Table 3. Preoperative and postoperative radiological characteristics of PPECD-VBD were shown in Fig. 4. AD, HD, CA, and SA were 5.62 ± 2.62, 0.17 ± 0.08, 18.67 ± 8.42, and 4.79 ± 1.44 points before surgery, respectively; and 5.90 ± 2.71, 0.19 ± 0.09, 19.17 ± 9.13, and 5.01 ± 1.43 points after surgery. There were no significant differences before and after surgery (*P* = 0.095, 0.100, 0.091, and 0.160, respectively). PPECD-VBD treatment did not cause segmental kyphosis, and had no significant effect on cervical curvature and stability.

**Table 3:** Radiological evaluation of VBD group

	Preoperatively	1 day Postoperatively	6 months Postoperatively	12 months Postoperatively	P value^a^
AD	5.62 ± 2.62	–	–	5.90 ± 2.71	0.095
HD	0.17 ± 0.08	–	–	0.19 ± 0.09	0.100
CA	18.67 ± 8.42	–	–	19.17 ± 9.13	0.091
SA	4.79 ± 1.44	–	–	5.01 ± 1.43	0.160

^a^ Between each item values of preop and postop 12 months in VBD group

## Discussion

At present, PECD is considered as an ideal minimally invasive decompression technique for the treatment of CSR [26–28]. However, the main target disease of PECD is still CSR caused by “soft” disc herniation, and the main decompression scope is still indirect dorsal decompression and removal of the free nucleus pulposus, without dealing with ventral hyperplasia of bone and ligaments in the surgical field [19–22].

In fact, CSR caused by cervical intervertebral foramen and lateral spinal stenosis combined with “hard” compression of the ventral side of the cervical nerve root and dural sac [29, 30], such as vertebral body posterolateral osteophytes, hook vertebral joint osteophytes, disc calcification, and ligament hyperplasia, which might be accompanied by “soft” disc herniation. Obviously, PPECD-SDD treatment cannot effectively remove the above substances; in other words, incomplete decompression. Oh Hyeong Seok et al. [25] reported that 12 (11.9%) of 101 CSR patients who received PECD experienced poor clinical efficacy; five patients had severe CFa/oLSS, and seven patients underwent an anterior approach to remove nerve ventral osteophytes again. This indicated that incomplete ventral decompression may be an important factor leading to poor postoperative curative effects of PECD. The present study found similar results. One patient in the SDD group relapsed 2 years after surgery, one patient still had a weak arm, and three other patients had no relief in pain of the upper limbs and neck after surgery. Among the three patients, one patient recovered following ACDF surgery to remove the ventral osteophytes after 3 months. The postoperative excellent and good rates in the VBD group were significantly better than those of the SDD group. This evidence demonstrated the importance of removing pressure vessels such as ventral osteophytes. Direct decompression of the ventral side was a key point in the treatment of CSR caused by CFa/oLSS.

**Fig. 5:**
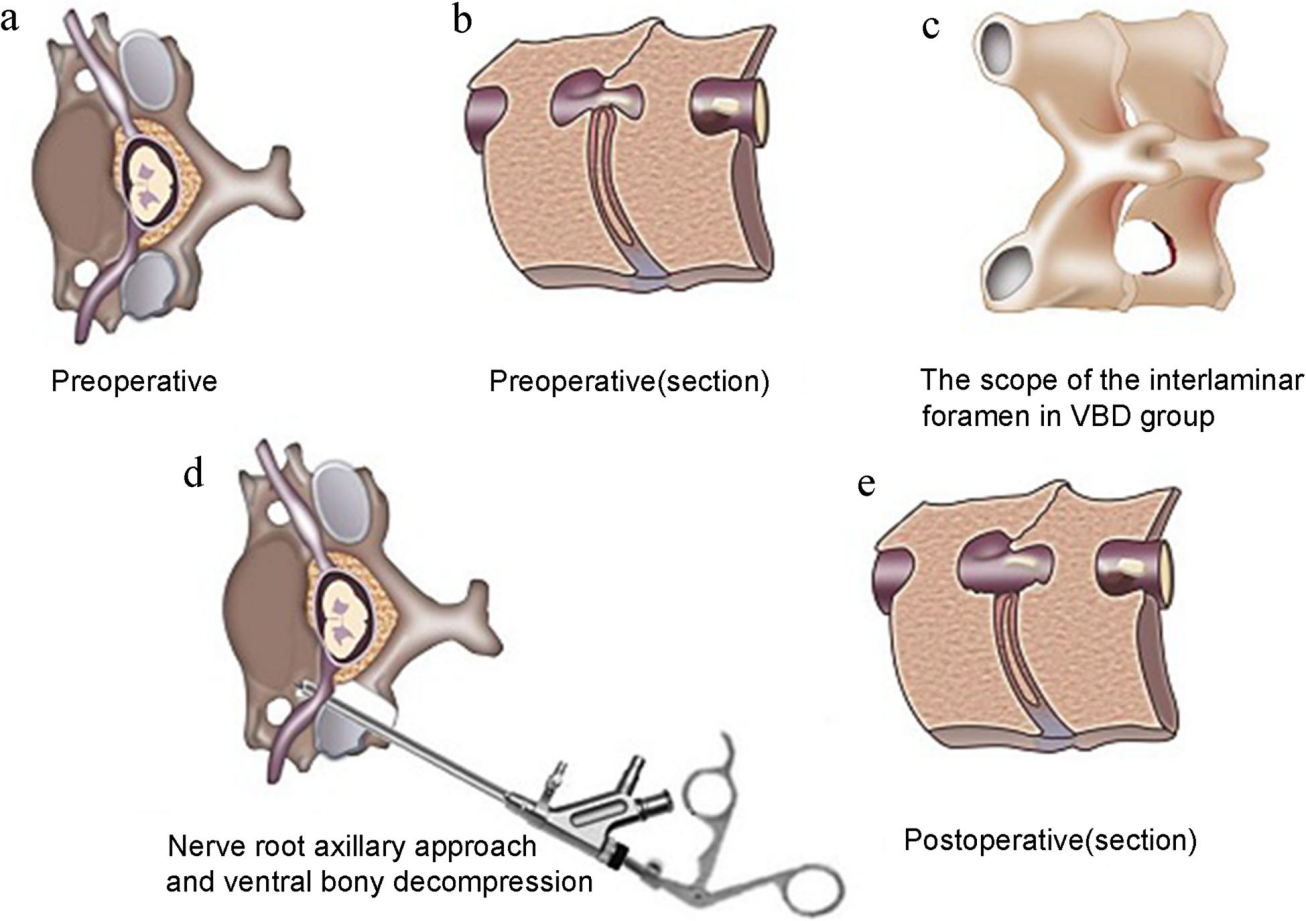
Model diagram for PPECD-VBD

To the best of our knowledge, the treatment of CSR caused by CFa/oLSS through traditional PECD (PPECD-SDD) has been seldom reported [23, 31]. We have summarized a large amount of clinical practice experience, improved and optimized surgical techniques, and achieved PPECD-VBD through a percutaneous dorsal approach. In fact, PPECD-VBD treatment is not significantly more difficult compared with PPECD-SDD for the reason that PPECD-VBD was developed from PPECD-SDD. Therefore, for the first time, the PPECD-VBD theory of posterior cervical percutaneous endoscopic surgery is proposed. The main technical points include: (1) The scope of the interlaminar foramen was slightly expanded outward and the tail side on the basis of the PPECD-SDD treatment, which enabled the endoscope to have sufficient space and an appropriate angle to visualize the ventral side of dural sac and nerve root through the axillary part of the root. An enlarged field of view makes it easier to master this technique and achieve ventral decompression of the nerve root and the outer edge of the dural sac. (2) The working cannula and endoscope are placed behind the intervertebral foramen, to reach the nerve root axilla and the outer edge of the cervical spinal cord at a certain abduction angle, and thus make it easier to perform ventral decompression. (3) The application of local anesthesia and intravenous general anesthesia allows the patient to be conscious, and an increased pain threshold of the patient provides better feedback during PPECD-VBD, which would avoid further damage to the spinal cord and nerves. (4) Reasonable application of instruments, such as the arc osseous chisel, holmium laser, nucleus pulposus forceps, and bony gripper can efficiently excise ventral osteophytes, calcified discs, and hyperplastic ligaments. In order to further elaborate the points, we have specially made a model diagram (Fig. 5). In this retrospective study, we compared the curative effect of PPECD-VBD and PPECD-SDD. The VBD group had lower arm-VAS, neck-VAS, and NDI compared with the SDD group after 12 months of follow-up and the VBD group achieved better relief of root symptoms such as pain numbness. Moreover, the improvement rate of JOA was significantly higher than that of the SDD group at 1 day, 6 months, and 12 months after surgery. Although patients in VBD group were more satisfied with improvement of affected limb myodynamia than those in SDD group in the clinical follow-up, our results, which was not completely consistent with our expectation, showed that there was no statistical difference in myodynamia between two groups at 12 months after operation. The reason may be related to limited samples and myodynamia evaluation methods. Besides, CT results in the PPECD-VBD showed that ventral osteophytes were fully removed and the volume of the lateral spinal canal and intervertebral foraminal nerve root channel was significantly enlarged. Thus, the advantages of PPECD-VBD are obvious. It should also be noted that the excellent and good rates of the SDD group in this study was 60.87%, which was significantly lower than previously reported for PECD. This might be due to the different inclusion criteria of the study subjects. In previous reports of PECD, CSR is mainly caused by “soft” disc herniation, and thus when removing the free disc, decompression of the dorsal side can be achieved. However, in the present study, CSR was caused by CFa/oLSS, rather than pure disc herniation, and thus PPECD-SDD treatment cannot achieve a direct and sufficient decompression effect. Moreover, previous reports of unsatisfactory curative effects with PECD treatment were patients suffering from severe CFa/oLSS [25, 32], which again confirmed that such patients require PPECD-VBD.

The operation time in the VBD group was approximately 32 mins longer than that of the SDD group, which may be because PPECD-VBD treatment required more ventral decompression. Two cases of dorsal adventitia injury of the nerve root occurred in each of the two groups; both cases occurred while grinding the interlaminar foramen. No other complications such as spinal cord and nerve root injuries were found, indicating that the risk of nerve injury mainly lies in the dorsal osteotomy phase of the drill, and direct decompression of the ventral side did not cause additional damage. This further indicates that two treatments are comparable in terms of security. PPECD-VBD treatment through the percutaneous endoscopic approach of the cervical spine requires expansion of the scope of resection of facet joints and laminectomy. The facet joint and laminectomy range play an important role in the development of segmental kyphosis after cervical foramen incision [33, 34]. Al-rawahi et al. [35] described a similar re-stabilizing effect of proliferating osteophytes on the spine. This study also investigated the effects of PPECD-VBD treatment on cervical spine curvature and stability. Postoperative follow-up CT showed that the average facet joint grinding degree of the VBD group was 38%. We analyzed cervical spine X-rays before and after surgery and found that there was no statistically significant change in cervical lordosis and stability in the VBD group. This is consistent with previous reports of PECD treatment [36].

This study still had some limitations, such the limited amount of clinical data collection and limited follow-up time, and the design of the study was limited to retrospective study. The medium-long-term curative effects and complications of PPECD-VBD required further study and exploration. Besides, this novel surgical technique is preferably implemented in very experienced hands.

## Conclusion

Based on the above results, the following conclusions can be drawn. Firstly, PPECD-VBD has significant effectiveness and safety in the treatment of CSR caused by CFa/oLSS. It also has no adverse effect on cervical curvature and stability. Besides, the clinical efficacy and patients’ satisfaction of PPECD-VBD is obviously better than PPECD-SDD. Above all, the recection of ventral osteophytes and hyperplastic ligaments has an important significance in the treatment of CSR caused by CFa/oLSS by posterior cervical percutaneous endoscope.

## Data Availability

The datasets used and/or analysed during the current study are available from the corresponding author on reasonable request.

## Abbreviations

PPECD-VBD: Posterior percutaneous cervical endoscopic decompression-ventral bony decompression
PPECD-SDD: Posterior percutaneous cervical endoscopic decompression-simple dorsal decompression
PCPE: Posterior cervical percutaneous endoscope
CFa/oLSS: Cervical foraminal and/or lateral spinal stenosis
PECD: Percutaneous endoscopic cervical decompression
CSR: Cervical spondylotic radiculopathy
CT: Computed tomography
VAS: Visual analogue scale
NDI: Neck disability index
JOA: Japanese Orthopedic Association Scores
AD: Angular displacement
HD: Horizontal displacement
SA: Segmental angle
CA: Cervical curvature
